# Editorial: Working towards sustainable agriculture with biostimulants and biocides: from chemical structure to application methods

**DOI:** 10.3389/fpls.2024.1443658

**Published:** 2024-07-31

**Authors:** Ágnes Szepesi, Cristiano Soares, Fernanda Fidalgo

**Affiliations:** ^1^ Department of Plant Biology, Institute of Biology, Faculty of Science and Informatics, University of Szeged, Szeged, Hungary; ^2^ GreenUPorto - Sustainable Agrifood Production Research Centre/Inov4Agro, Department of Biology, Faculty of Sciences, University of Porto, Porto, Portugal

**Keywords:** biocides, biostimulants, sustainable agriculture, allelochemicals, allelopathy, abiotic stress

Within the concept of “One Health”, which recognises that global sustainability will only be attained if humans, wildlife and ecosystems are equally preserved and taken care of, the transition of current agricultural practises towards more environmentally-friendly, yet efficient, approaches are a matter of special interest, especially for Agronomists, Plant Biologists and Breeders. Aligned with this vision, this Research Topic (RT) was designed to gather the latest findings on the development of bio-based compounds with potential application under a real agricultural scenario. Four original articles, published in this RT, provide excellent examples of sustainable approaches to enhance the efforts of eco-friendly agriculture by applying biostimulants and biocides.

Although natural herbicides could be a good alternative to synthetic herbicides, in order to be used in a safe and efficient manner, extensive screening is still needed, namely in the search for innovative bioactive compounds. A good example of this purpose is the work of Alanaz et al., which investigated the bio-herbicidal potential of several wild plant species collected from Tabuk region, in Saudi Arabia. The phytochemical screening of these wild species uncovered the presence of multiple allelochemicals, providing promising candidates for sustainable weed control schemes. For instance, the cultivation of these allelopathic-producing crops could be used in integrated weed management in agricultural food production systems.

On a different perspective, besides promoting plant growth and increasing crop abiotic stress tolerance, plant growth-promoting bacteria (PGPR) could also act as an effective tool for biological control against pathogenic microbes. Koo et al. revealed that YM002, identified as a strain of *Paenibacillus tianmuensis*, showed antagonistic activity against *Acidovorax citrulli*, the causal agent of bacterial fruit blotch, which induces severe diseases in cucurbit crops. The authors also evaluated various plant growth-related traits, suggesting that this PGPR is effective not only in suppressing the virulence of *Acidovorax citrulli* but also in helping the plants surviving to these harsh conditions.

Approaches targeting the improvement of crop growth are a current hot topic of research, given the need to maximize yields while reducing the footprint of chemical fertilizers. Within this perspective, soil amendments with organic materials or seed priming with elicitor molecules can be two complementary solutions with practical relevance. In this RT, the work conducted by Li et al. provides additional information on the underlying mechanisms behind chitin-mediated improvement of soil quality, plant growth and stress resilience. Upon soil amendment with chitin (derived from crab shells), authors monitored the growth of lettuce (*Lactuca sativa* L.), and followed the early transcriptional reprogramming and related metabolic changes. Data pointed to the upregulation of flavonoid-related pathways and to the downregulation of lignin biosynthesis. Metabolomics showed that some phenolic compounds, such as salicylic acid and chlorogenic acid, were influenced in chitin-treated lettuce seedlings, promoting plant defence networks. This work provides evidence of the effectiveness of chitin soil amendments in activating stress tolerance mechanisms, by priming lettuce plants and promoting growth via transcriptional changes.

Also aimed at increasing plant stress resilience, the study by Gao et al. focuses on new ways to alleviate salinity-induced effects in alfalfa (*Medicago sativa* L.). As soil salinization is an increasing problem, especially due to poor water management and inadequate irrigation practices, rising attention is being paid to the development of feasible mitigation methods, aimed at reducing agricultural costs and employing sustainable strategies. In this context, seed priming, namely with nanomaterials, has been standing out in recent years, though different efficacy rates appear to be dependent on the employed methodology. Gao et al. investigated the potential of CeO_2_NP seed priming on alfalfa plants under salt stress, by testing different concentrations of the priming agent; based on their data, a dose of 50 mg/L was optimal to improve the seed vigour, enhance the activity of α-amylase, regulate the osmotic balance and maximize endogenous hormone levels to improve salt tolerance of alfalfa seedlings. Still, further research is needed to decipher the background mechanisms of nanopriming in order to use them in a sustainable, safe and efficient manner.

Altogether, the articles published in this RT clearly demonstrate that agricultural systems can be improved in many different ways, especially when looking at nature-based solutions. By benefiting from plant growth-promoting microorganisms, altering soil structure and properties with organic amendments, and/or exploring plant endogenous resources to find new elicitor compounds, crop yield and stress resilience can be enhanced, and new pest and disease management approaches can be developed. Nevertheless, additional research is needed from the lab to the field, evaluating not only the efficiency of such strategies, but also their environmental safety, by assessing potential non-target effects on agroecosystems.

## Author contributions

ÁS: Conceptualization, Writing – original draft, Writing – review & editing. CS: Conceptualization, Writing – original draft, Writing – review & editing. FF: Conceptualization, Writing – original draft, Writing – review & editing.

